# Association between social capital and sleep duration among rural older adults in China

**DOI:** 10.1186/s12889-021-12441-w

**Published:** 2022-01-05

**Authors:** Le Yang, Hongman Wang, Jingmin Cheng

**Affiliations:** 1 School of Management, Shanxi Medical University, 56 Xinjian South Road, Taiyuan, Shanxi Province China; 2 School of Health Humanities, Peking University, 38 Xueyuan Road, Haidian, Beijing, China

**Keywords:** Social capital, Sleep duration, Rural older adults, China

## Abstract

**Objective:**

Sleep disturbances are great challenges to older adults’ health promotion. The study tested gender differences in the association between different dimensions of social capital and self-reported sleep duration of Chinese rural older adults.

**Design:**

The data of rural older adults were extracted from a national cross-sectional survey of the Chinese Longitudinal Healthy Longevity Survey (CLHLS) and analyzed in this study.

**Setting:**

CLHLS covered 23 provinces in China.

**Participants:**

The 6552 rural respondents aged ≥65 years old were involved.

**Main outcome measures:**

Generalized trust, informal social participation, formal social participation and social support were used to assess social capital. Self-reported sleep duration was measured as health outcome.

**Results:**

Low level of generalized trust had harmful effect on insufficient sleep (AOR 1.110, 95% CI 1.018-1.324), and having no formal or informal social participation was significantly positively associated with long sleep (AOR_formal_ 1.424, 95% CI 1.007-2.013; AOR_informal_ 1.241, 95% CI 1.016-1.516). Rural older female adults with no emotional social support had higher odds of insufficient sleep (AOR 1.502, 95% CI 1.258-1.978). Meanwhile, both informal and formal social participation showed inverse association with long sleep for females.

**Conclusions:**

This study found the relationship between social capital, sleep duration and the gender differences in Chinese rural older adults. More targeted sleep disturbance interventions could be taken in social capital of rural older adults, and gender differences should be considered when making social capital-embedded health promotion policies and interventions.

**What is already known on this subject:** Sleep has gradually become a focus of old adults’ health research for its impact on health. Numerous studies have suggested that social capital may be associated with sleep.

**What does this study add:** This study aimed to address the research questions that if there were connection between social capital and sleep duration and its gender differences in Chinese rural older adults.

## Introduction

Sleep has gradually become a focus of old adults’ health research for its impact on health. Sleep disturbances are great challenges for the older adults’ health promotion and sleep disorders are common in older adults for their decreasing ability to maintain sleep as the age increases [1]. With regards to sleep duration, there is a U-shaped association whereby short sleep is associated with health risks, as is long sleep, such as depression, obesity, cognitive impairment, and cardiovascular disease [2–5]. While sleep problems are always discussed and explained by age, there are many other factors accompanying aging, and sleep problems in older adults could be explained in multifactorial path. Many factors in the aging process, including physical and psychological conditions, changes in socio-economic environment and lifestyle, may cause sleep problems in older adults [6].

Among the social factors affecting sleep, social capital has been confirmed to be related to the sleep disturbances. Social capital refers to the association between individuals or groups, i.e. social networks, reciprocity, norms, trust and participation, and people could obtain resources and help from their social capital. The current approaches in social capital are mainly divided into two dimensions, social cohesion, and social network. Social cohesion places more emphasis on social capital at the macro-level, such as social trust, reciprocity and norms, while the social network is mainly concerned with micro-level social capital, such as the density and scope of an individual’s social network [7, 8]. There are many different perspectives in social capital measures, including cognitive (individuals’ perceptions, values, beliefs, and attitudes) and structural (externally observable social interactions), bonding (relations between people of groups of similar social identity), bridging (relations between people of groups of different social identity) and linking (formal relations to people of power and authority) [9–11]. Robbins (2019) found that short and long sleep duration were inversely related to measures of social capital, such as group memberships and a sense of neighborhood belonging, in adults in the Philadelphia area [12]. Another study showed poor neighborhood social capital was associated with insufficient sleep duration among rural Japanese adults [13]. In China, the association between social capital and sleep has also been studied, and previous researches presented that higher level of social capital is associated with higher sleep quality [14, 15]. However, few studies explored the association between social capital and sleep duration in China.

Due to the differences in age, gender, nationality, socioeconomic status, living environment, people have access to and rely on different sources and forms of social capital, thereby forming different means by which social capital affects health and different health outcomes. Gender difference, an important way to explain gender differences in health, has to rural social capital and sleep. Sleep disturbances increase with age in both males and females. While men and women sleep differently, women always typically report poorer quality and more disrupted sleep across various stages of life [16]. In addition, gender differences in forms of social capital and source of social connection would help to explain the gender difference in sleep disturbances [17–19]. Basset and Moore (2014) found the association among restless sleep, social capital, and neighborhood environmental factors differed in genders in Montreal adults [20]. In China, older adults with high-frequency of social engagement may have better sleep quality, but the positive relation between different types of social engagement and sleep quality was different between male and female [21]. Given the results of studies on gender differences in the association between social capital and health outcomes were inconsistent, the gender differences in the association between sleep duration and social capital remains to be examined.

Many studies have confirmed the differences in social capital between rural and urban elderly residents [22, 23]. China is a typical *guanxi*-based society, and *guanxi* in China refers to the personal connections or the special bonds in interpersonal networks, which are enduring, reciprocal, and sentimentally based instrumental relations [24–26]. Chinese tend to seek for social support through the guanxi and maintain the interpersonal network in their social life [27]. The traditional “*guanxi*” culture takes root more deeply in the rural area, and evidence shows “*guanxi*” and social capital have similar connotations and effects [28]. Previous studies show that bonding social capital comprising a connection between community members is often stronger among rural older adults, resulting in higher level of interpersonal trust, reciprocity and community cohesion [29, 30]. With the rapid urbanization and changes in family structure, older adults in rural areas need to face more obstacles to get care and support from family members (i.e., sons and daughters) who have increasingly migrated to urban areas for education and work opportunities [31, 32]. Some of them relocate to the town or city with their family members, which would affect the social capital of rural older adults, thereby affecting their health. Rural older adults predominate the old population in China, while to our knowledge, there are few studies focusing on association between social capital and rural older adults’ sleep duration. Given the concerning relationship between sleep duration and social capital, questions still remain unanswered regarding the underlying pathways between sleep duration and social capital among rural older adults in China.

Accordingly, in the present study the association between social capital and self-reported sleep duration in Chinese rural older adults were tested using a national sample of China. This study aimed to address the research questions that if there were connection between social capital and sleep duration and its gender differences in Chinese rural older adults.

## Methods

### Study design and population

Chinese Longitudinal Healthy Longevity Survey (CLHLS) covered 23 provinces and is the largest set of survey data of the old adults in China [33]. CLHLS conducted population surveys with 15,874 individuals in 2017-2018 (wave 8), including rural and urban areas, using a domiciliary face-to-face questionnaire, in the countries’ native language. In this study, those whose Hukou status (household registration) were rural and aged ≥65 were recognized as rural older adults. This study excluded respondents who were urban residents (*n* = 4477), under 65 years old (*n* = 74), lacked sleep duration information (*n* = 579), or provided implausible sleep duration of < 2 or > 16 h per day [34] (*n* = 81). The respondents who did not answer all the questions were regarded as unreliable and were excluded from the analysis, among them, 991 individuals missed social capital related information, 2877 individuals did not have complete sociodemographic data, and 243 individuals did not provide health related information. A final total of 6552 respondents were analyzed.

### Measures

#### Social capital

In health research on social capital and health outcomes, due to the different choices of social capital measures, the research results are also various. The results of research reports focusing on the relationship between collective social capital and health are not that clear, while social capital measures in individual-level is mostly found to be closely related to positive health outcomes [35]. This research was mainly concerned with the impact of individual-level social capital on sleep disturbances of old adults. This study combined elements of different approaches to capture individual-level social capital, such as generalized trust, social participation, and social support, which were shown as important social capital measures associated with health changes and healthcare utilization [36–38]. Social capital was captured using five variables measured: generalized trust (representing cognitive social capital); informal social participation and formal social participation [39] (representing structural social capital); emotional and instrumental social support (representing output).

1. Question about generalized trust: Do you feel that people around you are trustworthy? The variable response was classified into 1= trustworthy, 0= untrustworthy.
2. Question about social participation: (1) informal social participation: Do you visit and interact with friends regularly? The variable response was divided into 1= yes, 0=no; (2) formal social participation: Do you take part in social activities (organized) regularly? The variable response was divided into 1= yes, 0=no.
3. Question about social support: (1) Is there anyone that you could talk to when you need to tell something of your thoughts? (emotional social support); (2) Is there anyone you could ask for help when you have problems/difficulties? (instrumental social support). The variable response was grouped into 1= yes, 0=no.

#### Sleep variables

The average sleep duration per day of a participant was obtained using the question “How many hours do you sleep normally every day including napping?”, with respondents giving an integer number. Given the review of current related studies [40, 41], this study defined the sleep duration of 7-9h per day as the normal sleep duration for old adults, <7h per day as insufficient sleep, and > 9h per day as long sleep.

#### Covariates

This study selected the following as potential confounders according to the previous studies [12, 13, 42]. The first group of potential confounders were demographic and socioeconomic characteristics, including age, gender, current residential area, education (assessed as years of schooling), pension status (with pension vs. without pension), living alone (yes vs. no), marital status, and annual household income. Current residential area was classified as city, town, and rural area. Years of schooling was divided into 0 (uneducated), 1-6 years (primary school), 7-12 years (middle school and high school), and ≥13 years (college or above). Marital status was grouped as “married and living with spouse, separate, divorced, widowed, never married”. Household annual income was categorized as follows: low (≤10,000 yuan), medium (10,000-36,000 yuan), high (>36,000yuan). The second group consisted of variable of health behaviors, including current smoking (yes vs. no), current drinking (yes vs. no), and physical activity (yes vs. no). Lastly, covariates of variables of health condition were examined, such as sleep quality, body mass index (BMI), depressive symptoms, activities of daily living (ADL), cognitive function, noncommunicable disease (NCD), and comorbidity. The measure of self-reported sleep quality was dichotomized into very good/good versus other (fair/bad/very bad), and was treated as a binary outcome. The 10-item Center for Epidemiologic Studies Short Depression Scale (CES-D) was adopted to measure the depression symptom, scoring from 0 to 30 [43, 44]. ADL was based on whether the respondent could be independent in feeding, bathing, dressing, toilet hygiene, continence and indoor transferring, each item was scored from 1 to 3 (1 score representing complete independence; 2 scores representing partially dependence; 3 scores representing complete dependence), and lower scores indicating better functional independence. Cognitive function was measured using the Chinese version of Mini-Mental State Examination (MMSE), with a score ranging from 0 to 30 [45].

### Statistical analysis

This study was conducted to explore the association of social capital and self-reported sleep duration as well as its gender difference in the rural older adults. The continuous variables were described using mean and standard deviation (SD), and the categorical variables were presented by number and proportion (%). Given that the sampling weight variable in the CLHLS dataset was estimated based on the estimated numbers of older adults by age, sex, and rural/urban residence and there were no other important compositional variables, this study did not apply sample weights in the regression analyses, but the sampling weights were applied in the descriptive analysis and comparative analysis. Several studies have suggested that including variables related to sample selection in the regression produces unbiased coefficients without weights [46] and weighted regression results are likely to enlarge the standard errors [47].

To clarify the effects of social capital on sleep duration, a stepwise approach was used to adjust for different sets of confounding variables. Preliminary logistic regression model (model 1) was generated by applying merely the social capital variables to assess the association of social capital and sleep duration. In model 2, the study further adjusted for demographic and socioeconomic characteristics, in model 3, the study adjusted for demographic and socioeconomic characteristics, and health behaviors, and in model 4, the study adjusted for all confounders. Participants with sleep duration of 7-9h were set as the reference groups. Results were presented as ORs, 95% CI and corresponding P values. To examine gender differences in the association of social capital and sleep duration, we performed stratified analyses by gender group, adjusting for all confounders.

All statistical analyses were performed by SPSS ver. 24.0 (IBM, Armonk, NY, USA). The significance level was at two-tailed *P*-value <0.05.

## Results

The demographic and socioeconomic characteristics of respondents were presented in Table 1, most rural older adults (61.25%) lived in rural areas currently, but some lived in other areas (35.98% in town and 2.77% in city). There were 52.4% of participants reporting insufficient sleep (sleep duration <7h every day), and a small number of older adults (18.3%) experienced long sleep.

**Table 1:** Characteristics of the old adults by sleep duration

	<7h	7-9h	>9h	*p* value
Gender, N (%)				<0.001
Male	1647(40.1%)	1150(50.0%)	680(47.4%)	
Female	2461(59.9%)	1151(50.0%)	756(52.6%)	
Age, mean (SD)	81.77(11.24)	81.75(11.28)	87.19(11.32)	<0.001
Household annual income, N (%)				<0.001
≤10000	1871(45.5%)	895(38.9%)	571(39.8%)	
10000-36000	997(24.3%)	618(26.9%)	359(25.0%)	
>36000	1240(30.2%)	788(34.2%)	506(35.2%)	
Having pension				<0.001
Yes	334(8.1%)	266(11.6%)	120(8.4%)	
No	3774(91.9%)	2035(88.4%)	1317(91.6%)	
Education				<0.001
Uneducated	2132(51.9%)	1063(46.2%)	823(57.3%)	
Primary school	1577(38.4%)	887(38.5%)	504(35.1%)	
Middle school and high school	375(9.1%)	321(13.9%)	102(7.1%)	
College or above	24(0.6%)	31(1.3%)	8(0.5%)	
Live alone				<0.05
Yes	121(2.9%)	45(2.0%)	47(3.3%)	
No	3987(97.1%)	2256(98.0%)	1389(96.7%)	
Marital status, N (%)				<0.001
Married and living with spouse	1923(46.8%)	1136(49.4%)	496(34.5%)	
Separated	76(1.9%)	42(18%)	12(0.8%)	
Divorced	10(0.2%)	0(0.0%)	8(0.6%)	
Widowed	2071(50.4%)	1100(47.8%)	903(62.9%)	
Never married	27(0.7%)	23(1.0%)	17(1.2%)	
Current residential area, N (%)				0.332
City	123(3.0%)	64(2.8%)	30(2.1%)	
Town	1497(36.4%)	808(35.1%)	517(36.0%)	
Rural	2487(60.6%)	1429(62.1%)	889(61.9%)	
Smoking, N (%)				<0.01
Yes	687(16.7%)	459(19.9%)	263(18.3%)	
No	3421(83.3%)	1842(80.1%)	1174(81.7%)	
Alcohol drinking, N (%)				<0.001
Yes	586(14.3%)	405(17.6%)	261(18.2%)	
No	3522(85.7%)	1896(82.4%)	1175(81.8%)	
Physical activity, N (%)				<0.01
Yes	1173(28.6%)	738(32.1%)	383(26.7%)	
No	2935(71.4%)	1563(67.9%)	1054(73.3%)	
Having NCD, N (%)				<0.01
Yes	2804(68.2%)	1563(67.9%)	905(63.0%)	
No	1305(31.8%)	738(32.1%)	532(37.0%)	
Comorbidity, N (%)				<0.01
Yes	1500(36.5%)	837(36.4%)	460(32.0%)	
No	2608(63.5%)	1464(63.6%)	977(68.0%)	
Sleep quality, N (%)				<0.001
Good/very good	1260(30.7%)	1649(71.7%)	1141(79.5%)	
Other	2848(69.3%)	652(28.3%)	295(20.5%)	
ADL score, mean (SD)	6.66(1.99)	6.51(1.72)	6.71(1.75)	<0.01
MMSE score, mean (SD)	24.86(6.19)	25.16(6.04)	23.61(6.72)	<0.001
Depression score, mean (SD)	6.84(3.15)	7.31(2.79)	7.43(3.08)	<0.001
BMI, mean (SD)	25.40	27.88	29.57	<0.01
Generalized trust, N (%)				<0.001
Trustworthy	2919(71.1%)	1750(76.0%)	1065(74.1%)	
Untrustworthy	1189(28.9%)	552(24.0%)	372(25.9%)	
Social participation (informal), N (%)				<0.001
Yes	2706(65.9%)	1514(65.8%)	782(54.4%)	
No	1402(34.1%)	787(34.2%)	655(45.6%)	
Social participation (formal), N (%)				<0.001
Yes	461(11.2%)	267(11.6%)	100(7.0%)	
No	3647(88.8%)	2034(88.4%)	1336(93.0%)	
Social support (emotional), N (%)				0.169
Yes	4015(97.7%)	2234(97.0%)	1406(97.8%)	
No	93(2.3%)	68(3.0%)	31(2.2%)	
Social support (instrumental), N (%)				0.010
Yes	4048(98.5%)	2280(99.1%)	1429(99.4%)	
No	60(1.5%)	21(0.9%)	8(0.6%)	
Cases, N (%)	4108(52.4%)	2301(29.3%)	1437(18.3%)	

*P* values are calculated with analysis of Chi-squared test for categorical variables and variance (ANOVA) for continuous variables

Approximately 44.3% of the respondents were male and 55.7% were female. The proportions of female, without pension, uneducated, and widowed were relatively higher among those who reported insufficient sleep and long sleep (*P*<0.01). The similar proportions of the participants having NCD were observed among short and normal sleepers and lower proportion among long sleepers (*P*<0.01). The mean age was relatively older among those who had long sleep (*P*<0.001). A higher percentage of older population with low household annual income reported insufficient sleep (*P*<0.001). Meanwhile, older adults of normal sleep and long sleep showed lower percentage of non-smoking and non-drinking, and the proportion of those who did no physical exercise was higher among long sleepers. The mean score of ADL was relatively higher among those who suffered insufficient sleep and long sleep (*P*<0.001), and the mean score of depression symptom was higher among the long-sleep older adults. The rural older adults in China had higher social trust, more informal social activity involved, higher social support, but less formal social participation.

Multinominal logistic regressions on grouped sleep duration as a dependent variable were carried out (Table 2). Across all models, a significant association was found between generalized trust and insufficient sleep after controlling all potential confounders. Generalized trust had protective effect on insufficient sleep, older adults feeling people around them untrustworthy showed greater odds of short sleep in Model 1[Odds Ratio (OR) 1.237, 95% Confidence Interval (95% CI) 1.088-1.407], and the harmful effects were shrunk after controlling all the confounders in Model 4 [Adjusted Odds Ratio (AOR) 1.110, 95% CI 1.018-1.324]. Meanwhile, formal social participation was significantly inversely associated with long sleep. The older adults who did not participate in organized social activities had greater odds of long sleep in Model 1 (OR 1.678, 95% CI 1.275-2.208). After adjusting confounders (gender, age, education, marital status, current residential area, pension status, living alone, household annual income, smoking, alcohol drinking, physical activity, sleep quality, depression, BMI, ADL, cognition function, NCD and commodity), the harmful impacts were attenuated in Model 4 (AOR 1.424, 95% CI 1.007-2.013). The older adults who did not have informal social participation had greater odds of long sleep in Model 1 (OR 1.604, 95% CI 1.387-1.855), but the association was attenuated when variables of health condition (Model 4) were added.

**Table 2:** Logistic regression analysis of the association between social capital and sleep duration

		Model 1 (Crude OR)	Model 2^a^	Model 3^b^	Model 4^c^
<7h	Generalized trust				
	Untrustworthy	1.237** (1.088-1.407)	1.227** (1.078-1.397)	1.226** (1.077-1.396)	1.110* (1.018-1.324)
	Informal social participation				
	No	0.986 (0.875-1.112)	0.978 (0.862-1.109)	0.967 (0.851-1.098)	0.930 (0.783-1.106)
	Formal social participation				
	No	1.021 (0.849-1.228)	0.946 (0.783-1.143)	0.928 (0.768-1.123)	1.017 (0.792-1.305)
	Social support (emotional)				
	No	0.730 (0.503-1.059)	0.677 (0.465-0.986)	0.682 (0.468-0.994)	0.590 (0.350-0.994)
	Social support (instrumental)				
	No	1.395 (0.756-2.573)	1.418 (0.764-2.629)	1.432 (0.771-2.657)	2.240 (0.868-7.110)
>9h	Generalized trust				
	Untrustworthy	1.046 (0.889-1.231)	1.089 (0.923-1.285)	1.086 (0.921-1.282)	1.052 (0.851-1.300)
	Informal social participation				
	No	1.604*** (1.387-1.855)	1.241** (1.063-1.449)	1.244** (1.064-1.454)	1.241* (1.016-1.516)
	Formal social participation				
	No	1.678*** (1.275-2.208)	1.336** (1.010-1.769)	1.342** (1.012-1.778)	1.424* (1.007-2.013)
	Social support (emotional)				
	No	0.767 (0.477-1.234)	0.668 (0.411-1.084)	0.664 (0.409-1.078)	0.556 (0.295-1.047)
	Social support (instrumental)				
	No	1.013 (0.448-2.288)	1.185 (0.519-2.706)	1.178 (0.516-2.694)	2.077 (0.680-6.344)

Results are from proportional odds models. Results are displayed as ORs of change in sleep duration (taking 7-9 hours as reference) per unit increase in the original scale of generalized trust, visits and interaction with friends, participation of organized social activities, emotional social support, or instrumental social support

^a^ Adjusted for gender, age, education, marital status, current residential area, household annual income, living alone, and pension status

^b^ Adjusted for gender, age, education, marital status, current residential area, household annual income, living alone, pension status, smoking, alcohol drinking, and physical activity

^c^ Adjusted for gender, age, education, marital status, current residential area, household annual income, living alone, pension status, smoking, alcohol drinking, physical activity, sleep quality, BMI, depression, ADL, cognitive function, NCD, and comorbidity

*p < 0.05; **p < 0.01; ***p < 0.001

We further examined whether the association between social capital and sleep duration differed according to gender. As shown in Table 3, female older adults who had no emotional social support had higher odds of insufficient sleep (AOR 1.502, 95% CI 1.258-1.978), but males did not. At the same time, both informal and formal social participation showed inverse association with long sleep for females (i.e. people without social participation showed greater odds of long sleep).

**Table 3:** Logistic regression analysis of the association between social capital and sleep duration by gender

		Male	Female
<7h	Generalized trust		
	Untrustworthy	1.129 (0.872-1.461)	1.083 (0.848-1.384)
	Informal social participation		
	No	0.932 (0.724-1.199)	0.950 (0.747-1.207)
	Formal social participation		
	No	1.055 (0.756-1.472)	0.914 (0.621-1.346)
	Social support (emotional)		
	No	0.933 (0.412-2.113)	1.502* (1.258-1.978)
	Social support (instrumental)		
	No	3.317 (0.887-12.407)	2.145 (0.555-8.283)
>9h	Generalized trust		
	Untrustworthy	1.055 (0.776-1.434)	1.066 (0.793-1.434)
	Informal social participation		
	No	1.134 (0.848-1.518)	1.409* (1.065-1.864)
	Formal social participation		
	No	1.286 (0.827-2.002)	1.645* (1.028-2.634)
	Social support (emotional)		
	No	0.716 (0.247-2.070)	0.511 (0.229-1.140)
	Social support (instrumental)		
	No	2.392 (0.498-11.491)	1.634 (0.306-8.719)

Results are displayed as ORs of change in sleep duration (taking 7-9 hours as reference) per unit increase in the original scale of generalized trust, visits and interaction with friends, participation of organized social activities, emotional social support, or instrumental social support

Adjusted for gender, age, education, marital status, current residential area, household annual income, living alone, and pension, smoking, alcohol drinking, physical activity, sleep quality, BMI, depression, ADL, cognitive function, NCD, and comorbidity

*p < 0.05; **p < 0.01; ***p < 0.001

## Discussion

Using data from CLHLS-across-sectional survey, this study provided evidence for an association between social capital and sleep duration and its gender differences among Chinese rural older adults. This study found that generalized trust had protective effect on short sleep, as well as social participation (informal and formal) on long sleep, after controlling all potential confounders, but the association was modest. More female rural older adults reported short sleep or long sleep than males did. Having no emotional social support was associated with higher odds of short sleep and having no social participation (informal and formal) was associated with higher odds of long sleep among females.

The study found that generalized trust was associate with insufficient sleep. Old adults who did not trust people around them showed greater odds of insufficient sleep. With low-level of trust, rural old adults are less likely to get sense of safety and health information from interaction with other people and collective action, therefore they are physically and mentally vulnerable and have a high possibility of social isolation. Some studies have claimed that sleep is more a psychological behavior and the stress-buffering effect of a trustful and safe social environment on falling asleep [48, 49]. When people feel stressed, they usually feel physical tension and mental stress, and are more sensitive to the sleeping environment, or they focus too much on sleep, which may influence their sleep quality and shorten their sleep duration [50]. Higher level of trust is conducive to stress relief, information (health related) contagion and health behavior modification, which could improve sleep quality by reducing anxiety and stress [14].

Social support, as one of the important social factors affecting physical and psychological health of older adults [51, 52], refers to a person’s perception of the availability of help or support from others through their social network [53]. Many evidences have showed a robust association between social support and favorable sleep outcomes [54, 55]. This study found that emotional social support played an important role in rural older women’s insufficient sleep. Women always show greater responses to sleep loss in terms of mood deficits [56, 57]. Women in China, especially rural older women, also face a greater burden of discrimination and social inequality [58] that might have contributed to the insufficient sleep in response to emotional fluctuation. Therefore, those policymakers of the sleep intervention measure for the rural older females need to pay more attention to their psychological factors and emotional needs, and give them more emotional social support. A study conducted in a Japanese town demonstrated that insufficient sleep was associated with all dimensions of neighborhood social capital in men and not in women [8], which was inconsistent with the results of this study. This may be due to the different social capital types and measurements. The study measured Chinese old adults’ social capital in generalized trust, social participation and social support, but it did not include the detailed neighborhood social capital.

Besides the significant effect of generalized trust and emotional social support, the results indicated the inverse association between having social participation and long sleep, which was also found among rural old women. Social participation could provide a sense of belonging and enable the old adults to better integrate into society through shared time and engagement in social activities with friends and members of community group, reduce the sense of solitude, buffer stress and make the old adults more positive in life, which promotes their sleep at night in turn [59]. Social participation could also promote sleep through physical status improvement (i.e. delay functional decline and preserved cognitive functions) and promotion of healthy behaviours [59]. In China, most men and women have different gender patterns of access to social capital, which may cause different effects of social capital on health. Chinese men are more engaged in their career, colleagues and friends, and it is more conducive for men to socialize in formal occasions and accumulate corresponding social capital. Given the traditional culture and social responsibility in China, rural women have less social participation, especially formal social participation, and higher risk of social exclusion [60–62]. Without adequate social participation, older people living in rural areas would suffer more from depression [63], which was found associated with higher risk of longer sleep duration and daytime napping in previous research [64, 65]. Differences in family responsibilities have led to significant gender differences in sleep outcomes, such as longer self-reported sleep in women [66].Women in Chinese society, especially in rural areas, always spend more time to give family support such as unpaid care responsibilities for grand-children and spouse [67], which makes their social capital more concentrated on their family and relatives [68]. And rural women with lower socio-economic statuses occupy the lowest participation in general leisure activities [69]. With the acceleration of urbanization, rural older women might migrate to a strange new environment for supporting their children in family chores and taking care of their grandsons/granddaughters for their children move to town or city to get better education or work opportunities [70], so they face many challenges in adapting to the new environment, such as social isolation, language barrier, loss of network and social interaction from the place of origin, and lack of resources to rebuild their social network [71–74], which leads to less social participation. Less social participation outdoor and spending more time doing family chores and taking care of their grandchildren at home, may give them more flexibility to nap or go to bed earlier [66]. Another reason could be the poor health condition, in other words, the higher dependence on ADL reduce their willingness and ability to interact with their friends and participate in organized social activities, causing longer sleep. It also needs to be noted that the potential dark side of the impact of social capital on rural older women’s health. Since rural older women have less formal social participation, the formal social participation may boost their stress [75], causing harmful effects on their health.

This study has some limitations. First, the analysis was conducted based on a cross-sectional data and did not find the cause-effect relationship between social capital and sleep duration, and the mediation mechanism of social capital on sleep in different genders needs to be further explored. Second, self-reported sleep duration every day (including napping) was used as the outcome variable, which may cause a possibility of recall bias considering that people often overestimate duration, but it is still as reliable as the objective assessments [76]. Some studies suggested sleep duration and daytime napping have similar association with certain health outcomes [77, 78], while some other studies found their dissimilarities [79], and future studies need to distinguish the two types of sleep in the association between social capital and sleep. Third, social capital in this study was not measured comprehensively, and different types of social participation were not all captured, for example, the older adults with insomnia were not included in the analysis due to lack of data. There were only a few cases having no emotional/instrumental social support, hence the accuracy of our estimation on the association between social capital and sleep duration would be compromised. Further study could examine how to improve social capital to promote healthy sleep and the mechanism of social capital’s impact on sleep in older adults from the perspective of gender differences, considering the different individual, community, society, and culture context.

## Conclusions

This study examined the relationship between social capital and sleep duration and found there were gender differences in the relationship of different dimensions of social capital and sleep disturbances in Chinese rural older adults. The results indicated the protective effect of generalized trust on insufficient sleep and the protective effect of informal and formal social participation on long sleep in rural older adults. For different genders, women tend to report more sleep problems than men, and there are also gender differences in the impact of social capital on sleep duration, suggesting that gender differences should be considered to promote healthier behaviors with regards to optimal sleep duration, especially the improvement of emotional social support and social participation among rural older women. Compared to the dietary or physical activity behaviour, sleep behaviour may be a more easily modifiable lifestyle factor and may therefore have significant potential to improve old adults’ health [80, 81]. Given the results, more targeted sleep disturbance interventions could be taken in social capital of rural older adults, and gender differences should be considered when making social capital-embedded policies and interventions to promote healthy sleep behaviours and better social environment.

## Data Availability

PKU Centre for Healthy Ageing and Development. 2021. “Chinese Longitudinal Healthy Longevity Survey (CLHLS).” Peking University Open Research Data. 10.18170/DVN/WBO7LK.
